# Integrative Taxonomic Approach for Describing a New Cryptic Species of Bush Frog (*Raorchestes*: Anura: Rhacophoridae) from the Western Ghats, India

**DOI:** 10.1371/journal.pone.0149382

**Published:** 2016-03-02

**Authors:** H. Priti, Rekha Sarma Roshmi, Badrinath Ramya, H. S. Sudhira, G. Ravikanth, Neelavara Anantharam Aravind, Kotambylu Vasudeva Gururaja

**Affiliations:** 1 Suri Sehgal Centre for Biodiversity and Conservation, Royal Enclave, Ashoka Trust for Research in Ecology and the Environment, Sriramapura, Jakkur Post Office, Bengaluru, India; 2 Manipal University, Manipal, India; 3 Research and Development Center, Gubbi Labs LLP, II Cross Extension, Gubbi, India; 4 Science Media Center, Gubbi Labs LLP, WS-5, I Floor, Entrepreneurship Center, Society for Innovation and Development, Indian Institute of Science Campus, Bengaluru, India; State Natural History Museum, GERMANY

## Abstract

A new cryptic species of bush frog *Raorchestes honnametti* sp. nov. is described from the south-eastern part of the Western Ghats, India. This newly described species belongs to the *Charius* clade and is morphologically similar to other clade members—*R*. *charius* and *R*. *griet*. Therefore, an integrative taxonomic approach based on molecular and bioacoustic analysis along with morphology was used to delimit the new species. *Raorchestes honnametti* sp. nov., is currently known only from Biligiri Rangaswamy Temple Tiger Reserve, a part of Biligiri Rangaswamy horst mountain range (a mountain formed due movement of two faults) formed during the Late Quaternary period (1.8–2.58 Ma). Discovery of cryptic species from a highly speciose and well-studied genus *Raorchestes* hints at the possible existence of several more cryptic species in this genus. We discuss the possible reasons for crypsis and emphasize the need for continued systematic surveys of amphibians across the Western Ghats.

## Introduction

Cryptic species are morphologically similar and difficult to distinguish from their closest congeners [1]. Studies have shown that cryptic species are common and many of them go undetected when studied solely on the basis of morphological evidence. In recent years, molecular genetic techniques and bioacoustics have helped in the discovery of cryptic anurans [2, 3]. Identifying cryptic species is of paramount importance not only for biodiversity conservation but also to understand processes of evolution and biogeography [1, 4].

The Western Ghats of India is known for high diversity of amphibians with 220 species recorded. Along with high diversity, many studies have also suggested presence of high number of cryptic species in the Western Ghats. Using molecular techniques, Nair et al [5] carried out a study on endemic genus *Indirana*, and demonstrated high cryptic diversity among them. Similarly, two new genera in the family Rhacophoridae were discovered, in a cryptic genus *Polypedates* [6]. Using bioacoustics and molecular techniques, cryptic species were discovered from widely distributed genera like *Euphlyctis* (2 species, [7]) and *Fejervarya* (4 species, [8]).

The genus *Raorchestes* belongs to family Rhacophoridae. It includes bush frogs with adult size ranging from 10 mm to 50.5 mm. They are distinguished by the presence of transparent/translucent vocal sac and the absence of vomerine teeth. Adults are nocturnal and lack free swimming larvae [9]. There are 59 valid species in the genus *Raorchestes* [10], of which 50 species are from the Western Ghats, 4 from Eastern Himalaya, 1 from Eastern Ghats and 4 species from China, Myanmar and Vietnam. Bee et al [11] indicated the possible existence of several undescribed cryptic species in the genus *Raorchestes*. However, till date, there is no published literature about cryptic species from this genus. Here we describe a new cryptic bush frog species from south eastern region of the Western Ghats. The description is based on an integrative taxonomic approach involving morphology, molecular techniques and bioacoustics. We discuss the possible reason for cryptic speciation and the need for more detailed studies on cryptic species of Western Ghats.

## Materials and Methods

### Ethics statement

Fieldwork and sampling was carried out in the Biligiri Rangaswamy Temple Tiger Reserve (BRTTR) of Karnataka, with due permission from the Director, BRTTR dated 10 October 2012. Tissue sampling was carried out under the strict supervision of local foresters and used solely for scientific research. Minimum samples were collected (six individuals) as required for the study and the sampling do not in any way affect effect the population of this species. Field Permit issued by Principal Chief Conservator of Forests and Chief Wildlife Warden, Karnataka State Forest Department.

### Nomenclatural acts

The electronic edition of this article conforms to the requirements of the amended International Code of Zoological Nomenclature, and hence the new names contained herein are available under that Code from the electronic edition of this article. This published work and the nomenclatural acts it contains have been registered in ZooBank, the online registration system for the ICZN. The ZooBank LSIDs (Life Science Identifiers) can be resolved and the associated information viewed through any standard web browser by appending the LSID to the prefix “http://zoobank.org/”. The LSID for this publication is: urn:lsid:zoobank.org:pub:93347FEF-6BF8-40A2-9EEB-2F2C743E70FC. The electronic edition of this work was published in a journal with an ISSN, and has been archived and is available from the following digital repositories: PubMed Central, LOCKSS.

### Study area

**Fig 1:**
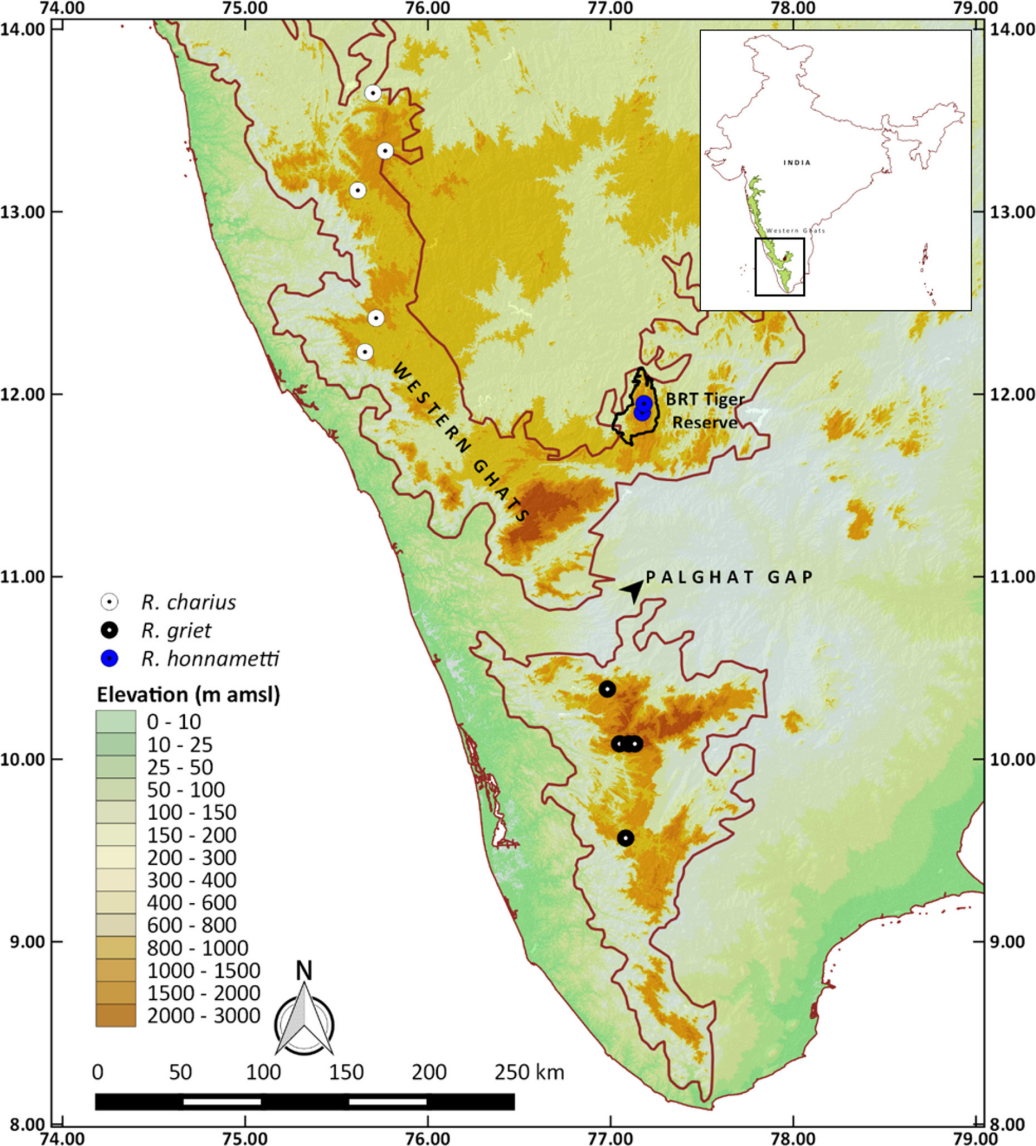
Biligiri Rangaswamy Temple Tiger Reserve and sampling sites of *Raorchestes honnametti* sp. nov. (blue circles). Maps were generated using QGIS® Pisa Ver. 2.10. Data was sourced from www.gadm.org for administrative boundary of India and Shuttle Radar Topography Mission (SRTM) 90 m database (http://srtm.csi.cgiar.org) for elevation.

**Fig 2:**
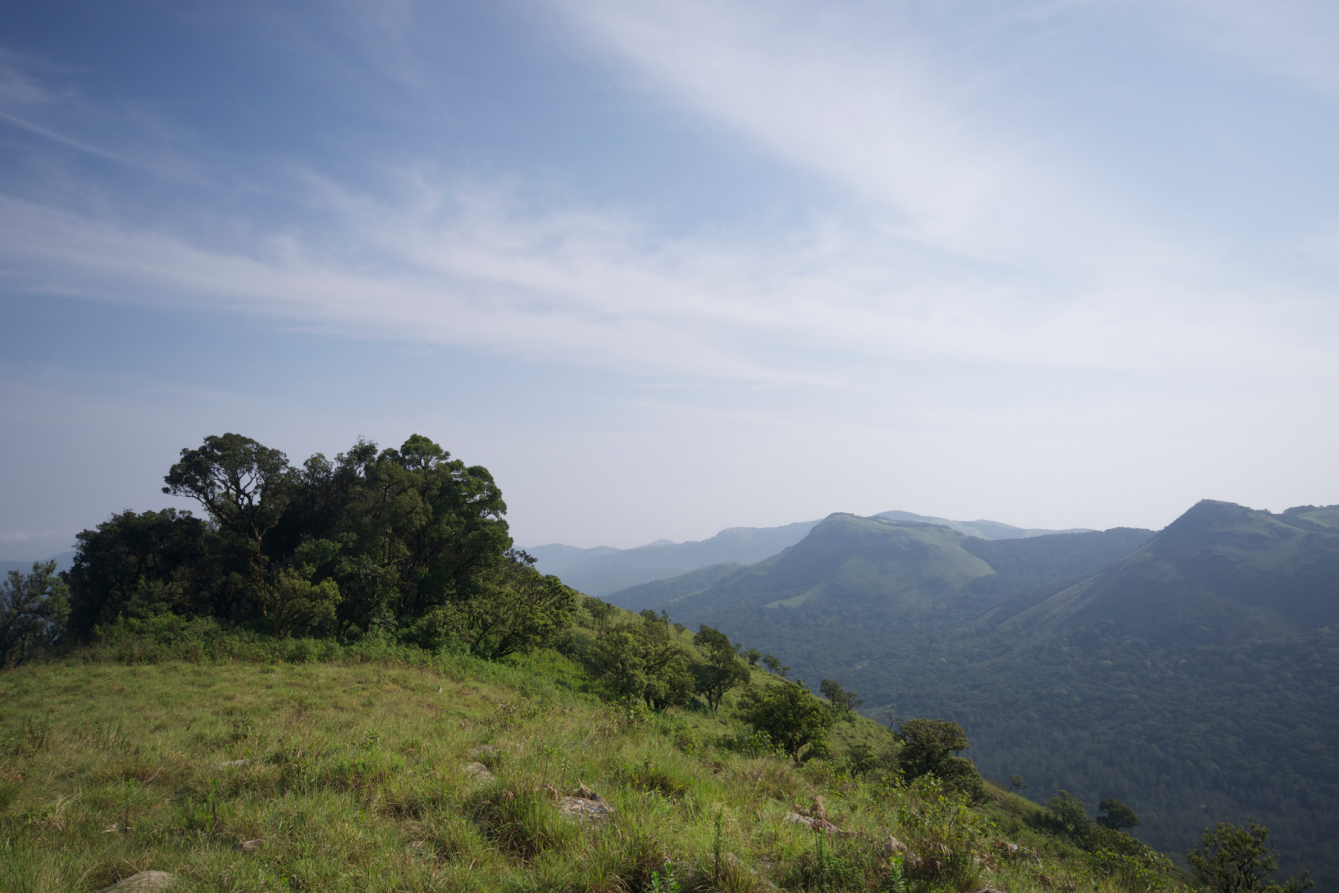
Type locality of *Raorchestes honnametti* sp. nov.–a high altitude shola forest at Honnametti in BRTTR.

The study was carried out in BRTTR (Biligiri Rangaswamy hill range; latitude: 11°–12.15°N, longitude: 77°–77.2667°E, altitude: 600–1800 m amsl). Specimens were collected from shola forests (shola forests are a type of forest in the valleys of a mountain separated by montane grasslands) in Honnametti (11.8987° N, 77.1741° E, 1659 m amsl) and Dodda Sampige (11.9473° N, 77.1836° E, 1142 m amsl) within BRTTR (Figs 1 and 2). The reserve got its name “Biligiri” from the white cliff on which the temple of Lord Rangaswamy is situated. This tiger reserve is located to the east of the Western Ghats in Karnataka State. The total area of the tiger reserve is 540 km^2^. The landscape is very heterogeneous and undulating. The annual rainfall, received mainly during the southwest monsoon, ranges from 600 mm at the base to 3000 mm at the hilltop. The variation in temperature ranges from 8°C to 25°C. The wide range of climatic conditions as well as altitudinal variations within the small area has resulted in varied forest types like scrub forest, dry deciduous, moist deciduous, riparian, evergreen, sholas and grasslands [12]. The extent of the major forest types represented as percent of total area is as follows: deciduous (moist and dry) = 61.1%, scrub = 28.2%, grassland = 3.4%, evergreen = 6.5% and high altitude sholas = 0.8% [13].

### Specimen collection

Six adult calling males were collected in October 2012. Specimens collected were euthanized and fixed in 5% formalin for 24h and preserved in 70% alcohol. Thigh muscle tissue samples for genetic analysis were collected before fixing the specimen. Color of live specimens and natural history notes were recorded at the type locality during multiple visits. Specimens were deposited in the Bombay Natural History Society (BNHS) museum, Mumbai.

### Genetic analysis

For DNA extraction, we followed the method described in Vences et al [14]. A small amount of thigh muscle tissue was excised from the holotype for extraction. The PCR amplification and sequencing of 16S rRNA and ND1 genes (subunit of NADH dehydrogenase) were done following Palumbi et al [15] and Bossuyt et al [16] respectively. The amplified products were sent to Amnion Sequencing services, Bangalore, India. The sequences were checked manually using program Chromas lite 2.01 (http://www.technelysium.com.au/chromas_lite.html). The sequences were aligned using MAFFT algorithm [17] and manually corrected in MEGA 5.10 software [18] and deposited in GenBank (Accession numbers, KT151650–KT151653). The final dataset consisted of 1032 base pairs. 16S and ND1 genes of 49 valid species of the genus *Raorchestes* endemic to the Western Ghats [19, 20, 21] were used in phylogenetic analysis. GenBank accession numbers for 16S rRNA and ND1 genes of 49 valid species used in the molecular analysis are given in S1 and S2 Tables, respectively. The phylogenetic analyses were performed using Maximum likelihood (ML) algorithm and Bayesian inference methods. The ML analysis was executed in raxmlGUI v1.3 [22] with GTR+G model selected as the best-fit nucleotide substitution model in jModel test [23] for 1000 bootstrap replicates. The Bayesian analysis was performed in MrBayes 3.2.4 [24]. The combined data set of 16S and ND1 partitioned as separate gene fragments were used for the analysis with TIM2+I+G and TPM3uf+I+G selected as best fit models for 16S and ND1 genes respectively in jModel test. The Markov chain Monte Carlo analysis for the dataset was run for 50 million generations and trees were sampled every 500 cycles. The convergence of the runs was analyzed by assessing the split frequency standard deviations (<0.001) and potential scale reduction factor (PSRF ~1.0). The first 10% of the sampled trees were discarded as burn-in and remaining samples were used to generate majority rule consensus tree. For estimating the genetic divergence, uncorrected pairwise genetic distance between the species was calculated in MEGA 5.10.

### Morphology

The new species was compared with all valid species of *Raorchestes* both from published literature and through examination of type specimens deposited in India (S3 Table). After the phylogenetic analysis, initial morphological comparisons were made with the members of the *Charius* clade. *Raorchestes thodai* [25] from Western Ghats lacked genetic data and was morphologically compared with the new species. Similarly, other non-Western Ghats species of *Raorchestes*, namely, *R*. *terebrans* from Eastern Ghats [26], *R*. *annandalii* [27], *R*. *manipurensis* [28], *R*. *sahai* [29] and *R*. *shillongensis* [30] from Eastern Himalayas; *R*. *gryllus* [31], *R*. *longchuanensis* [32, 33, 34], *R*. *menglaensis* [32, 35] and *R*. *parvulus* [36] from Myanmar, Vietnam and South-China were also compared morphologically with the new species. Morphological comparisons were made with synonymised species based on their original description, namely, *R*. *emeraldi* [21], *R*. *montanus* [37, 38, 39] and *R*. *neelanethrus* [40] to verify the availability of those names to the new species. Morphological measurements were taken using a Mitutoyo^®^ digital slide calliper to the nearest 0.1 mm. Measurement and terminology follows Biju and Bossuyt [20] and Gururaja et al [41]. Abbreviations used are as follows: snout–vent length (SVL); head width, at the angle of the jaws (HW); head length, from the rear of the mandible to the tip of the snout (HL); inter upper eyelid width, i.e. the shortest distance between the upper eyelids (IUE); maximum upper eyelid width (UEW); snout length, measured from the tip of the snout to the anterior orbital border of the eye (SL); eye length, i.e. the horizontal distance between the bony orbital borders of the eye (EL); internarial distance, i.e. least distance between the inner margins of nares (IN); nostril–snout distance, i.e. distance between middle of nostril and tip of snout (NS); eye to nostril distance, i.e. distance between anterior-most point of eye and middle of nostril (EN); tympanum–eye distance, i.e. anterior rim of tympanum to posterior of eye (TYE); distance from the rear of the mandible to the nostril (MN); distance from the rear of the mandible to the anterior orbital border of the eye (MFE); distance from the rear of the mandible to the posterior orbital border of the eye (MBE); distance between anterior corner of eyes, i.e. the shortest distance between the anterior orbital borders of the eyes (IFE); distance between posterior corner of eyes, i.e. the shortest distance between the posterior orbital borders of the eyes (IBE); largest tympanum diameter (TYD); forelimb length, measured from the elbow to the base of the outer palmar tubercle (FLL); hand length, measured from the base of the outer palmar tubercle to the tip of the third finger (HAL); thigh length (TL); shank length (ShL); foot length, measured from the base of the inner metatarsal tubercle to the tip of the fourth toe (FOL); distance from the heel to the tip of the fourth toe (TFOL); disc width on fingers I, II, III and IV (FD I, II, III and IV); width of finger I, II, III and IV measured at the base of the disc (FW I, II, III and IV); lengths of fingers I, II, III and IV measured from base of proximal subarticular tubercle to fingertip (FL I, II, III and IV); tibia width, i.e. width of tibia at its widest region (TW); disc width on toes I, II, III, IV and V (TD I, II, III, IV and V); width of toes I, II, III, IV and V (ToW I, II, III, IV and V) measured at the base of disc; length of toes I, II, III, IV and V measured from base of proximal subarticular tubercle to tip of toe (ToL I, II, III, IV and V); length of inner metatarsal tubercle (IMT); distance from distal edge of metatarsal tubercle to maximum incurvature of web between fourth and fifth toe (MTFF); distance from distal edge of metatarsal tubercle to maximum incurvature of web between third and fourth toe (MTTF); distance from maximum incurvature of web between fourth and fifth toe to tip of fourth toe (FFTF); distance from maximum incurvature of web between third and fourth toe to tip of fourth toe (TFTF). Intercalary ossification, which is the cartilaginous structure between distal and penultimate phalanges in fingers and toes was observed. The presence of an intercalary ossification can be noticed without anatomical sectioning as a glandular projection between phalanges [42]. We carried out linear discriminant analysis (LDA) using R v. 3.1.3 [43] and R Studio v. 0.98.1102 (http://www.rstudio.com/) on morphological data. The data was converted as ratio of SVL and tested for normality. Eight morphological characters viz., HW, HL, UEW, SL, HAL, TL, ShL and FOL were subjected to LDA to get a linear combination of these characters to discriminate *R*. *charius*, *R*. *griet* and *R*. *honnametti*. To identify multivariate differences among species MANOVA was performed and Tukey HSD post-hoc test was used for pairwise comparison of characters.

### Advertisement call recording and analysis

Call recordings were made using Olympus stereo digital voice recorder (LS-11) for *R*. *charius* and *R*. *honnametti*. Calls of *R*. *griet* were recorded using Canon EOS 600D. Calls with less background noise were manually selected from different individual call records and were analysed using Audacity Ver.1.3 (Beta) and Raven Pro 1.5. Nineteen calls from 3 individuals of *R*. *honnametti*; 20 calls from 4 individuals of *R*. *charius* and 8 calls from 1 individual of *R*. *griet* were selected for further analysis. Duration, inter-call interval duration, dominant frequency and number of pulses of each call was recorded. Call terminology was based on Kok and Kalamandeen [44]. Air temperature and relative humidity were recorded using TFA digital Thermo-Hygrometer. We used Welch t-test in R v.3.1.3 to compare the means of call characteristics between two species, assuming normal distribution of call samples with unequal variance and sample size.

## Results

### Genetic analysis

**Fig 3:**
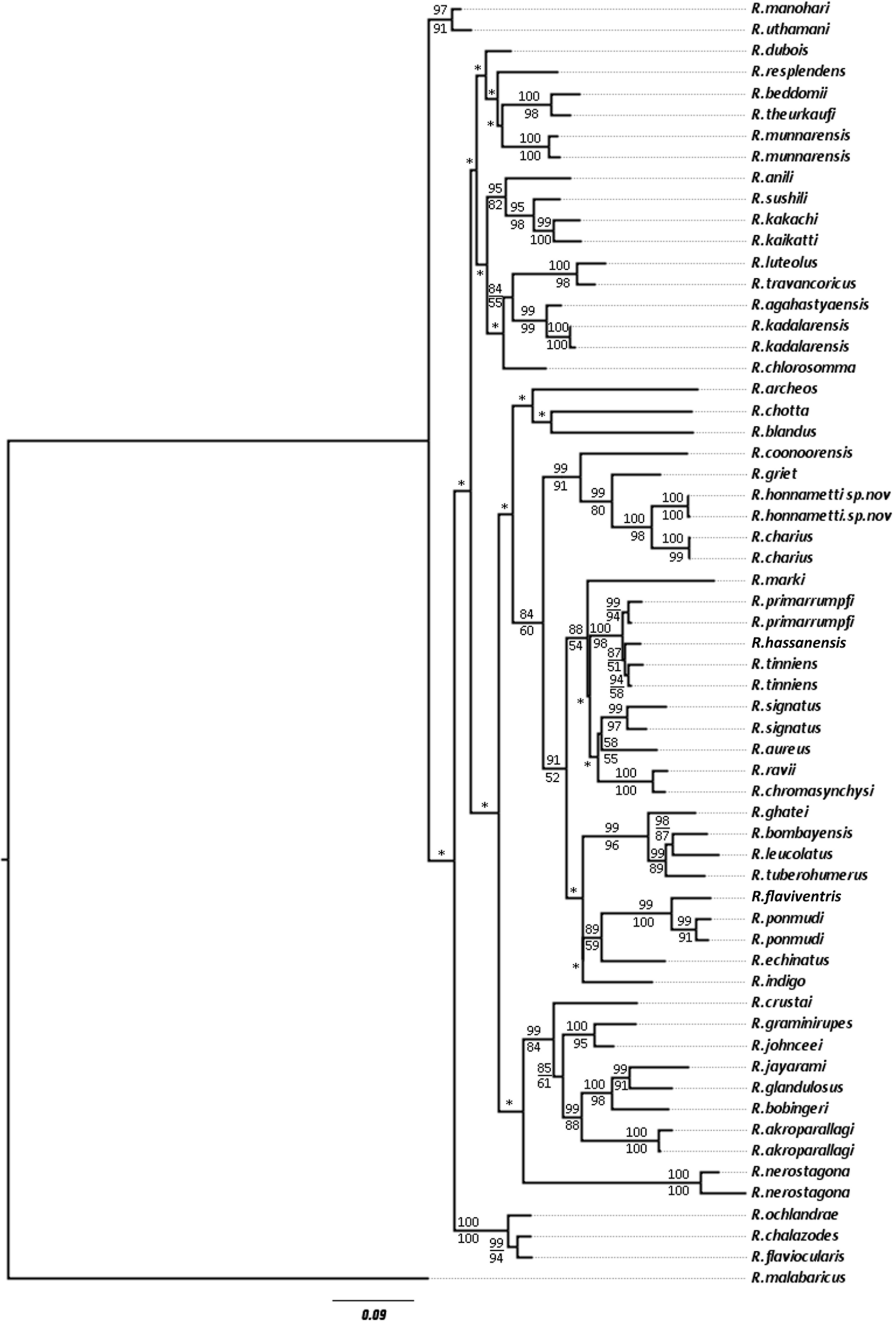
Maximum Likelihood tree for 50 *Raorchestes* species from the Western Ghats biodiversity hotspot and an outgroup *Rhacophorus malabaricus*. Numbers above and below the nodes indicate Bayesian Posterior Probabilities and Maximum Likelihood Bootstrap values >50, respectively. Asterisk (*) indicates values <50.

The molecular data analysis suggested that *Raorchestes honnametti* sp. nov. belonged to the ‘*Charius* clade’ [21] comprising of *R*. *charius* and *R*. *griet* The uncorrected pairwise genetic distance (Mean ± SD) between *R*. *honnametti* and *R*. *charius* is 4.14 ± 0.14% and between *R*. *honnametti* and *R*. *griet* is 6.47 ± 0.17%. Fig 3 shows the result of ML tree with a strong bootstrap support (100%) for the *R*. *honnametti* and *R*. *charius*. Hence, the morphological comparisons are made only with the closest relatives of the species, i.e. *R*. *charius* and *R*. *griet*. The overall genetic divergence of 50 species, including *R*. *honnametti* for the combined dataset of 16S rRNA and ND1 genes ranges from 2 to 32% (S4 Table).

### *Raorchestes honnametti* sp. nov. Gururaja, Priti, Roshmi and Aravind

urn:lsid:zoobank.org:act:071B913C-BB18-4E6A-97C6-6BA2621F8D6E

#### Suggested common name

Honnametti bush frog.

*Holotype*: BNHS 5941, an adult male collected by authors from *Strobilanthus* shrubs at 0.48m above ground at Honnametti, on 13^th^ October 2012 at 20:20 h from Biligiri Rangaswamy hills (11.8987° N, 77.1741° E, 1659 m amsl).

*Paratypes*: BNHS 5942, BNHS 5943, BNHS 5945 and BNHS 5946, male individuals collected by authors in Honnametti, collection date and place same as holotype. BNHS 5944, a male collected by authors on 14^th^ October 2012 at 19:45 h Dodda Sampige (11.9473° N and 77.1836° E, 1142 m amsl).

*Diagnosis*: *Raorchestes honnametti* belongs to the genus *Raorchestes* as they are relatively small sized frogs (15–45 mm), active in night, vomerine teeth absent, transparent/translucent vocal sac while calling and direct development without free swimming tadpoles. It is a small sized adult (male: 21.7–24.8 mm, n = 6); snout longer than the horizontal diameter of eye; groin uniform light brown with 3–4 yellow blotches; both anterior and posterior part of thigh uniform light brown with small round to oval shaped yellow blotches and relatively short hind limbs ShL/SVL ratio <0.5. It belongs to the *Charius* clade and morphologically similar to *R*. *charius* and *R*. *griet*.

**Fig 4:**
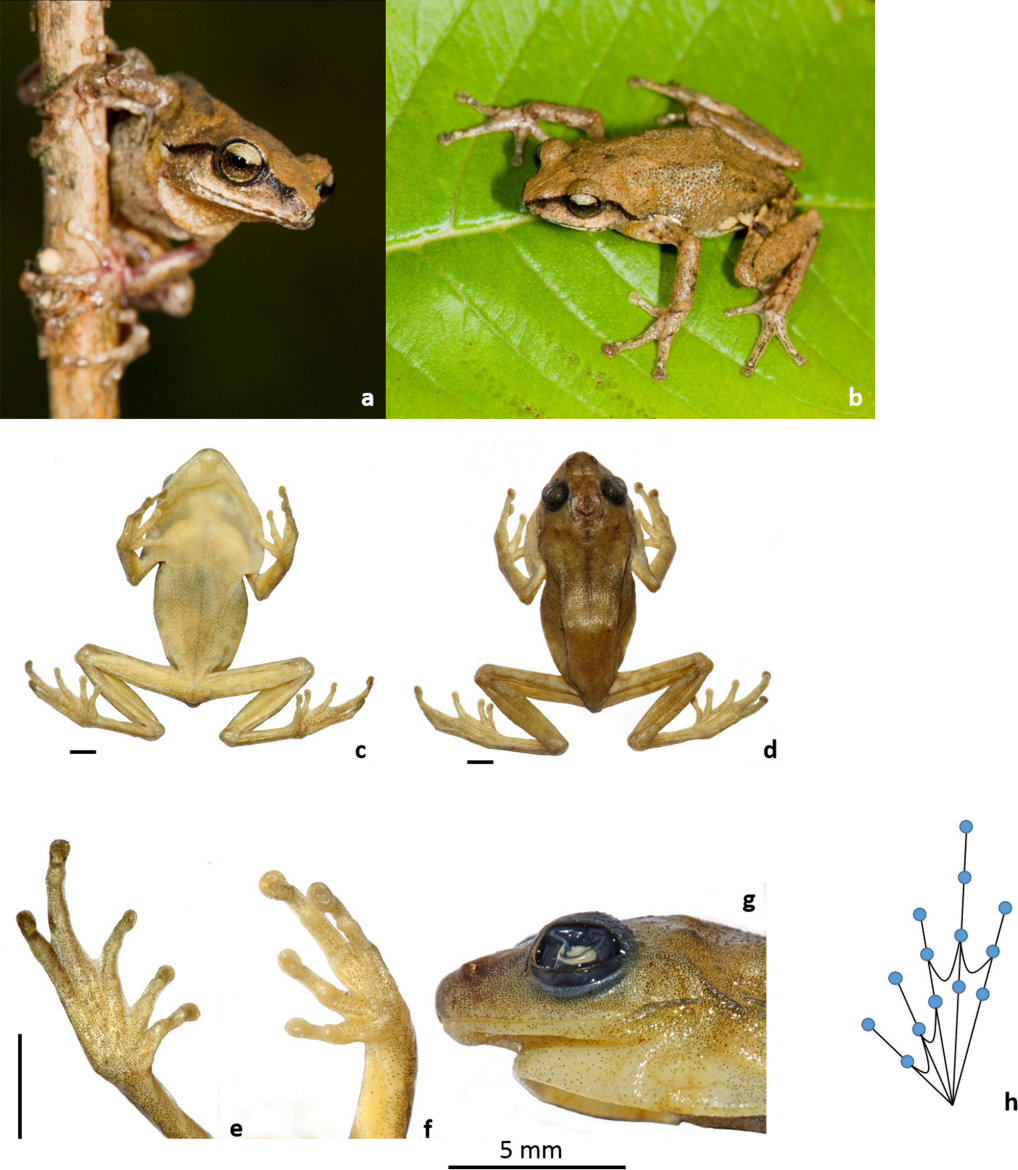
Holotype (BNHS 5941) of *Raorchestes honnametti* sp. nov. a-b—Live specimen; c—ventral view; d—dorsal view; e—ventral view of hind limb; f—ventral view of forelimb; g—lateral profile of head; h—Schematic view of webbing in hind limb.

*Description of holotype*: A small sized frog (SVL = 24, Fig 4A–4H, Table 1, all measurements in mm), head wider than long (HW = 8.0; HL = 7.4), snout oval in dorsal view and in profile rounded. Snout longer than or equal to eye (EL = 3.2; SL = 3.6). Canthus rostralis rounded, loreal region slightly concave. Interorbital space flat, almost equal to upper eyelid width and internarial distance (IUE = 2.5; UEW = 2.1; IN = 2.2). Internarial distance between posterior margins of eyes 1.7 times that of anterior margins (IFE = 4.3; IBE = 7.2). Nostrils oval, without flap, closer to tip of snout than to eye (NS = 1.4; EN = 2.0). Weak symphysial knob. Pineal ocellus absent. Tympanum indistinct, oval, closer to eye (TYD = 1.4), 2.3 times in eye length. Supratympanic fold distinct from back of eye to shoulder. Median sub-gular vocal sac with a pair of openings at the base of lower jaw. Tongue bifid, chordate, sparsely granular. Lingual papilla absent. Eyes moderately large (EL = 3.2), protruding, pupil horizontal.

**Table 1:** Morphometric data of *Raorchestes honnametti* sp. nov. (Holotype and paratypes). Measurements in mm.

**Characters**	**BNHS 5941 Holotype**	**BNHS 5942 Paratype**	**BNHS 5943 Paratype**	**BNHS 5944 Paratype**	**BNHS 5945 Paratype**	**BNHS 5946 Paratype**	**Mean ± SD**
Sex	Male	Male	Male	Male	Male	Male	
**SVL**	24.0	24.8	23.9	23.4	25.0	21.7	23.8 ± 1.2
**HW**	8.00	8.2	8.5	8.4	8.3	8.7	8.4 ± 0.2
**HL**	7.4	7.2	7.1	7.1	7.1	7.3	7.2 ± 0.1
**IUE**	2.5	3.2	3.2	3.5	3.2	3.6	3.2 ± 0.4
**UEW**	2.1	1.9	2.0	2.0	1.8	2.1	2.0 ± 0.1
**SL**	3.6	3.6	3.7	3.1	3.3	3.9	3.5 ± 0.3
**EL**	3.2	3.3	3.1	3.1	3.3	2.8	3.1 ± 0.2
**MN**	5.6	5.9	5.8	5.7	5.9	6.1	5.8 ± 0.2
**MFE**	6.0	4.6	4.5	4.5	4.3	4.4	4.7 ± 0.6
**MBE**	2.5	2.1	2.2	2.1	2.2	2.1	2.2 ± 0.2
**IN**	2.2	2.4	2.4	2.0	2.5	2.4	2.3 ± 0.2
**IFE**	4.3	4.2	4.0	4.1	4.3	5.3	4.4 ± 0.5
**IBE**	7.2	7.0	7.4	7.5	7.6	7.9	7.4 ± 0.3
**NS**	1.4	1.6	1.5	1.3	1.5	1.4	1.5 ± 0.1
**EN**	2.0	2.2	2.3	1.7	1.9	2.1	2.0 ± 0.2
**TYD**	1.4	1.7	1.2	1.3	1.6	1.1	1.4 ± 0.2
**FLL**	5.1	5.1	5.4	5.0	6.1	4.7	5.2 ± 0.5
**HAL**	7.1	7.6	7.5	6.8	7.1	6.8	7.2 ± 0.3
**FD1**	0.7	0.7	0.7	0.7	0.7	0.6	0.7 ± 0.0
**FD2**	0.9	0.9	0.7	0.9	0.9	0.8	0.9 ± 0.1
**FD3**	1.1	1.3	1.1	1.2	1.0	1.2	1.2 ± 0.1
**FD4**	1.2	1.1	1.0	1.2	1.1	1.0	1.1 ± 0.1
**FW1**	0.6	0.5	0.5	0.5	0.4	0.5	0.5 ± 0.1
**FW2**	0.4	0.5	0.5	0.6	0.6	0.5	0.5 ± 0.1
**FW3**	0.6	0.5	0.6	0.7	0.7	0.7	0.6 ± 0.1
**FW4**	0.6	0.6	0.5	0.6	0.7	0.6	0.6 ± 0.1
**FIL**	1.9	1.9	1.9	1.7	1.7	1.7	1.8 ± 0.1
**FIIL**	2.4	2.3	2.4	2.3	2.2	2.1	2.3 ± 0.1
**FIIIL**	3.2	4.4	3.8	3.6	4.5	3.9	3.9 ± 0.5
**FIVL**	2.3	3.1	3.6	3.1	3.9	3.2	3.2 ± 0.5
**FL**	11.3	11.5	11.4	10.3	11.9	11.9	11.4 ± 0.6
**ShL**	10.6	10.9	11.3	10.3	10.9	10.7	10.8 ± 0.3
**TW**	2.5	1.9	2.6	2.3	2.5	3.1	2.5 ± 0.4
**FOL**	10.2	10.6	10.9	8.8	10.3	9.8	10.1 ± 0.7
**TW1**	0.5	0.5	0.5	0.5	0.6	0.6	0.5 ± 0.1
**TW2**	0.5	0.5	0.5	0.6	0.5	0.6	0.5 ± 0.1
**TW3**	0.6	0.5	0.6	0.6	0.6	0.6	0.6 ± 0.0
**TW4**	0.7	0.6	0.6	0.6	0.8	0.7	0.7 ± 0.1
**TW5**	0.6	0.6	0.7	0.6	0.8	0.8	0.7 ± 0.1
**TD1**	0.8	0.7	0.7	0.8	0.8	0.6	0.7 ± 0.1
**TD2**	0.8	0.9	0.8	0.7	0.8	0.7	0.8 ± 0.1
**TD3**	0.8	0.7	0.9	0.9	0.7	0.7	0.8 ± 0.1
**TD4**	1.0	1.0	1.0	1.1	0.8	0.9	1.0 ± 0.1
**TD5**	1.0	1.1	1.0	1.1	0.8	1.0	1.0 ± 0.1
**IMT**	1.0	1.1	0.9	1.	0.9	0.8	1.0 ± 0.1
**TFOL**	16.4	11.1	17.5	15.1	16.9	16.2	15.5 ± 2.3
**TIL**	1.9	2.0	1.8	1.8	1.7	1.7	1.8 ± 0.1
**TIIL**	2.0	2.2	2.0	1.9	2.0	2.1	2.0 ± 0.1
**TIIIL**	3.2	3.3	3.3	3.0	3.2	3.0	3.2 ± 0.1
**TIVL**	5.3	5.5	5.6	4.4	6.3	5.0	5.4 ± 0.6
**TVL**	3.8	4.1	3.0	3.5	4.3	3.9	3.8 ± 0.5
**MTFF**	6.4	5.6	5.9	5.1	5.4	6.0	5.7 ± 0.5
**MTTF**	5.1	4.7	4.5	4.2	4.4	5.2	4.7 ± 0.4
**TFTF**	5.7	5.6	5.9	4.6	5.9	4.6	5.4 ± 0.6
**FFTF**	4.7	5.7	5.1	4.9	4.8	4.5	5.0 ± 0.4

Fore limb length shorter than hand length (FLL = 5.1; HAL = 7.1). Relative lengths of fingers I<II<IV<III (FL I = 1.9; FL II = 2.4; FL III = 3.2; FL IV = 2.5). Finger tips with well-developed discs (FD I = 0.7, FD II = 0.9, FD III = 1.1, FD IV = 1.2; FW I = 0.6, FW II = 0.4, FW III = 0.6, FW IV = 0.6) with circum marginal grooves, grooves fold upwards, giving bifid appearance in dorsal view, intercalary ossification distinct between penultimate and distal phalanges. Dermal fringe weak, on both sides of the fingers. Webbing between fingers absent. Subarticular tubercles distinct (finger: i = 1, ii = 1, iii = 2, iv = 1) rounded and pre–pollex tubercle oval, distinct. Supernumerary tubercles present. Nuptial pad absent.

Hind limbs long, do not overlap or touch when folded at right angles to body. Shank 4.2 times longer than wide (ShL = 10.6; TW = 2.5), shorter than thigh length (TL = 11.3) and longer than foot length (FOL = 10.0). Heel to tip of fourth toe (TFOL = 16.4) 3.1 times longer than fourth toe length (ToL IV = 5.3). Relative toe length I<II<III≤V<IV (ToL I = 1.9; ToL II = 2.0, ToL III = 3.2; ToL IV = 5.3; ToL V = 3.8). Toes with well develop discs at tip (TD I = 0.8, TD II = 0.8, TD III = 0.8, TD IV = 1.0, TD V = 1.0; ToW I = 0.5, ToW II = 0.5, ToW III = 0.6, ToW IV = 0.7, ToW V = 0.6). Webbing moderate (MTTF = 5.1, MTFF = 6.4, TFTF = 5.7, FFTF = 4.7). First toe (ToL I = 1.9) 1.9 times the length of inner metatarsal tubercle (IMT = 1.0). Outer metatarsal tubercle absent, supernumerary tubercles and tarsal tubercle present (toe: i = 1, ii = 1, iii = 2, iv = 3, v = 2).

*Skin*: Entire dorsum, snout and upper eyelid with very small horny spines, mid-dorsum dense with small horny spines; dorsal part of forelimbs, thighs and shanks smooth; throat, chest, belly, lower flanks and ventral sides of forelimbs and thighs granular; chest with larger granules; no dorso lateral fold,

*Colour in life*: Dorsum light grey with two dark grey concave stripes behind eyes to groin; light brown stripe between eyes demarcates triangular light grey snout, limbs barred, brown band from snout passing through loreal, mid iris and tympanic region till end of supratympanic fold. Iris golden above and light golden below, horizontally separated by brown band; dorsal side of forelimbs and hind limbs with dark grey cross bands. A triangular white spot on the tip of the snout. Venter uniform cream white, vocal sac yellow translucent with granular grey spots. Webbing cream white. Groin uniform light brown with 3–4 yellow blotches, both anterior and posterior part of thigh uniform light brown with small round to oval shaped yellow blotches.

*Colour in preservative*: Dorsum light grey, dorsal side of forelimbs and hind limbs with faint cross bands. Venter uniform cream white, vocal sac with granular grey spots. Webbing cream white. Groin uniform pale brown with 3–4 cream white blotches, both anterior and posterior part of thigh uniform pale brown with small round to oval shaped cream white blotches.

*Etymology*: Named after the locality of holotype–Honnametti. Honnametti is treated as an invariable noun in apposition to the generic name.

*Variation*: Details of the morphometric variations observed in 6 individuals is provided in Table 1. The dorsum colouration varied from light grey to dark brown. A triangular white spot on the tip of the snout are seen in couple of individuals. Flanks light grey to dark brown, yellow blotches in the groin varies both in numbers (2 to 5) as well as in size. Variations included distinct cross bands on both forelimbs and hind limbs.

**Fig 5:**
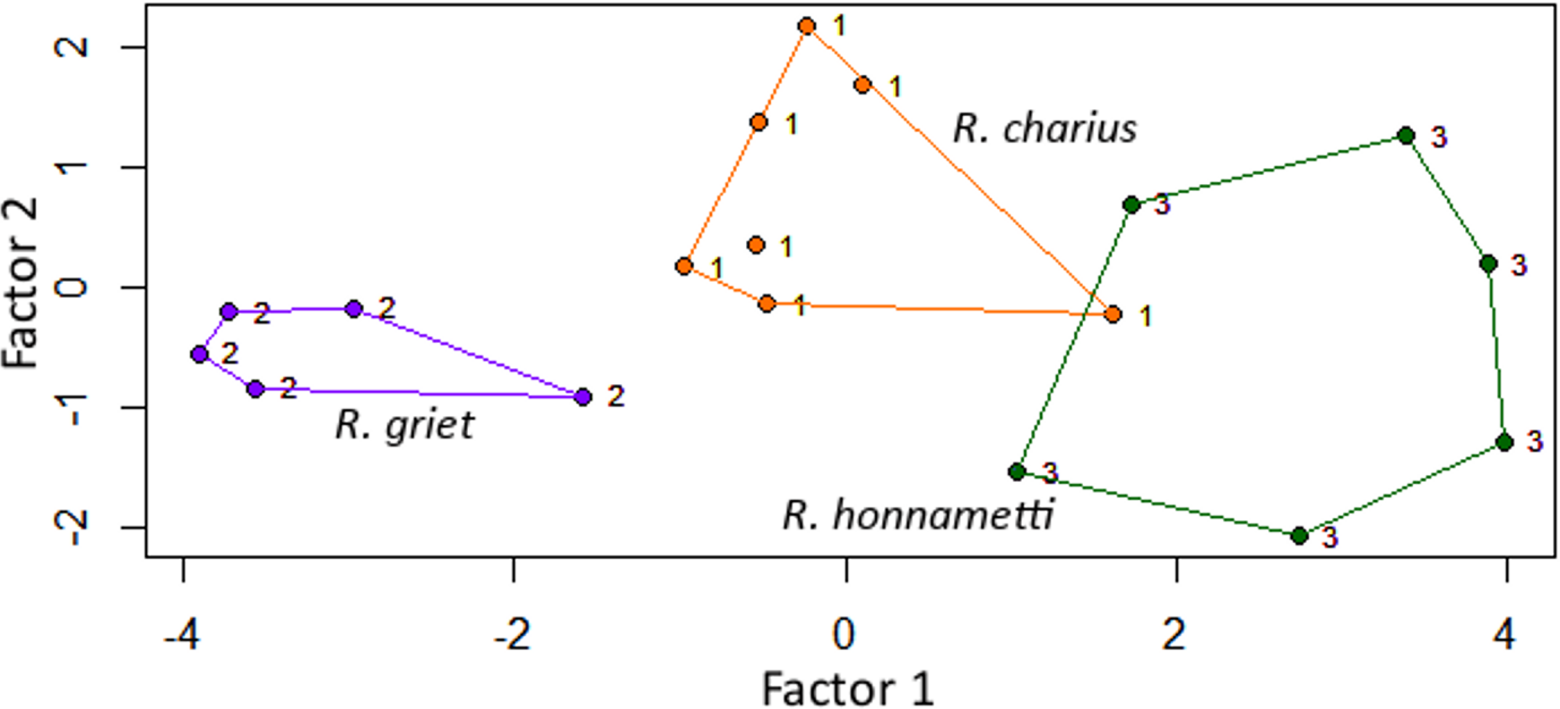
Linear discriminant analysis of morphometric data of the *Charius* clade members.

*Comparison*: Molecular data suggested that *Raorchestes honnametti* belonged to the *Charius* clade within the Western Ghat endemic species of *Raorchestes* and hence initial morphological comparisons were made only with *R*. *charius* and *R*. *griet*. *Raorchestes charius* differs from *R*. *honnametti* in following characters: adult size (*R*. *charius*: 27.2–31.4 mm vs *R*. *honnametti*: 21.7–24.8 mm), snout length (*R*. *charius*: 3.5–4.3 mm vs. *R*. *honnametti*: 3.1–3.9 mm) shorter or sub-equal to eye vs. snout length longer than eye (*R*. *charius*: 3.5–4.4 mm vs. *R*. *honnametti*: 2.8–3.3 mm), groin brownish black with yellow blotches vs. groin brown with 3–4 yellow blotches; posterior surface of thighs uniform brownish black with large yellow blotches vs. both anterior and posterior surface of thighs light brown with small round-oval shaped yellow blotches. *Raorchestes griet* differs from *R*. *honnametti* in following characters: adult size (*R*. *griet*: 19.7–22.4 mm vs. *R*. *honnametti*: 21.7–24.8 mm), snout length (*R*. *griet*: 2.4–2.7 mm vs. *R*. *honnametti*: 3.1–3.9 mm) shorter than eye vs. longer than eye (*R*. *griet*: 2.4–2.8 mm vs. *R*. *honnametti*: 2.8–3.3 mm), groin light brown with minute white marbling vs groin brown with 3–4 yellow blotches; posterior surface of thighs dark brown with some light brown spots vs. both anterior and posterior surface of thighs light brown with small round-oval shaped yellow blotches. Fig 5 shows LDA plot between three species. There is an overlap between *R*. *honnametti* and *R*. *charius*. *Raorchestes griet*, however, forms a distinct cluster. Factor loading for axis 1 and 2 are given in S5 Table. MANOVA did not result in significant differences between the three species (F_2,16_ = 1.61, p = 0.17). Tukey’s HSD post-hoc test also did not exhibit significant differences in pairwise comparison of characters except for SL in *R*. *griet* and *R*. *honnametti* (Q = 4.73, p = 0.012); TL in *R*. *griet* and *R*. *charius* (Q = 3.83, p = 0.041) and TL in *R*. *honnametti* and *R*. *griet* (Q = 5.03, p = 0.008).

Morphological comparisons of *R*. *honnametti* with *R*. *thodai* and with non-Western Ghats species of *Raorchestes* are given in S6 Table. *Raorchestes honnametti* differed from these congeners. Similarly, morphological comparison with synonymised species showed that earlier names were not available for the new species.

**Fig 6:**
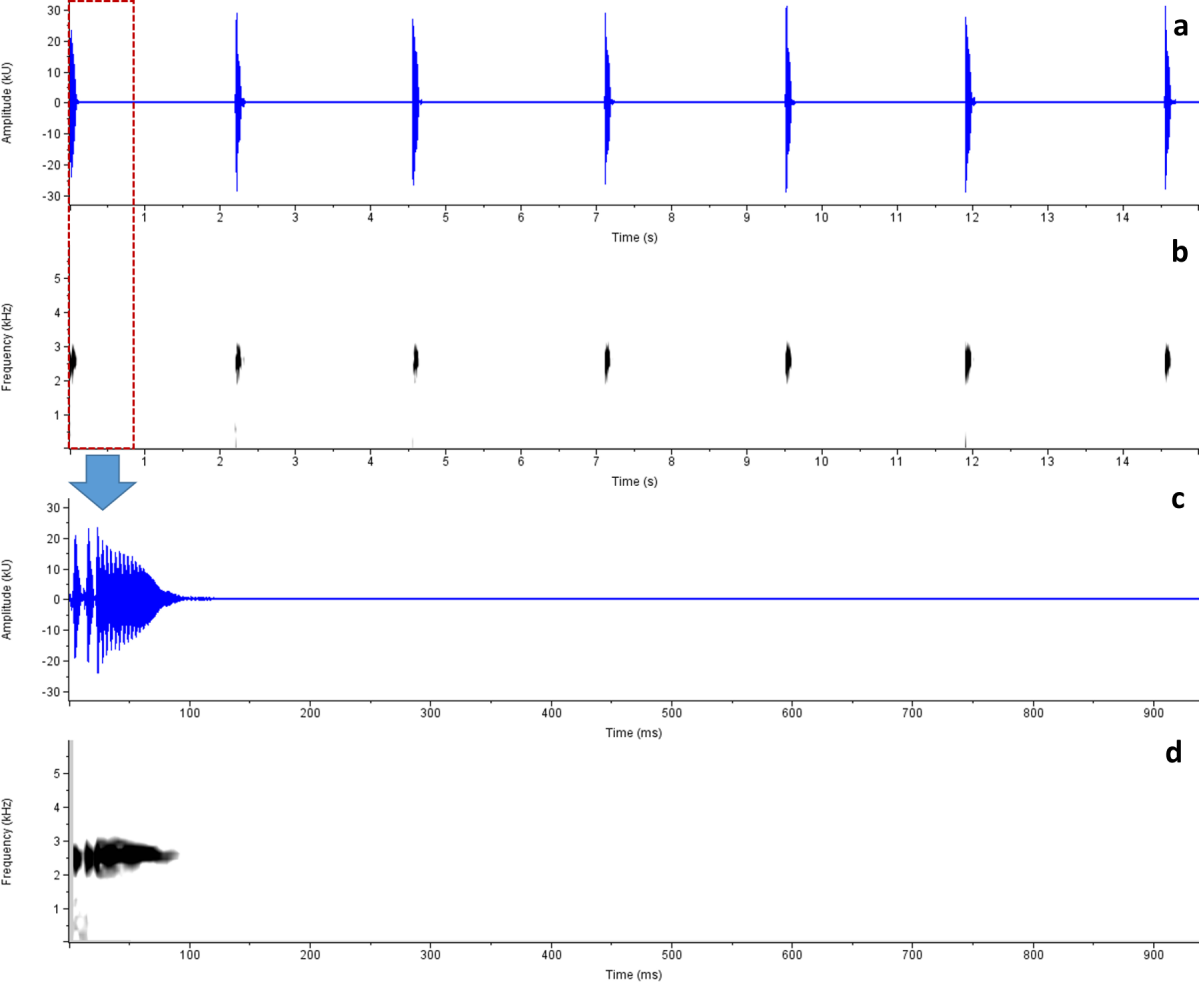
Advertisement call spectrogram of *Raorchestes charius*. a. Amplitude, b. Spectrogram, c. Amplitude of a single call and d. Spectrogram of a single call.

**Fig 7:**
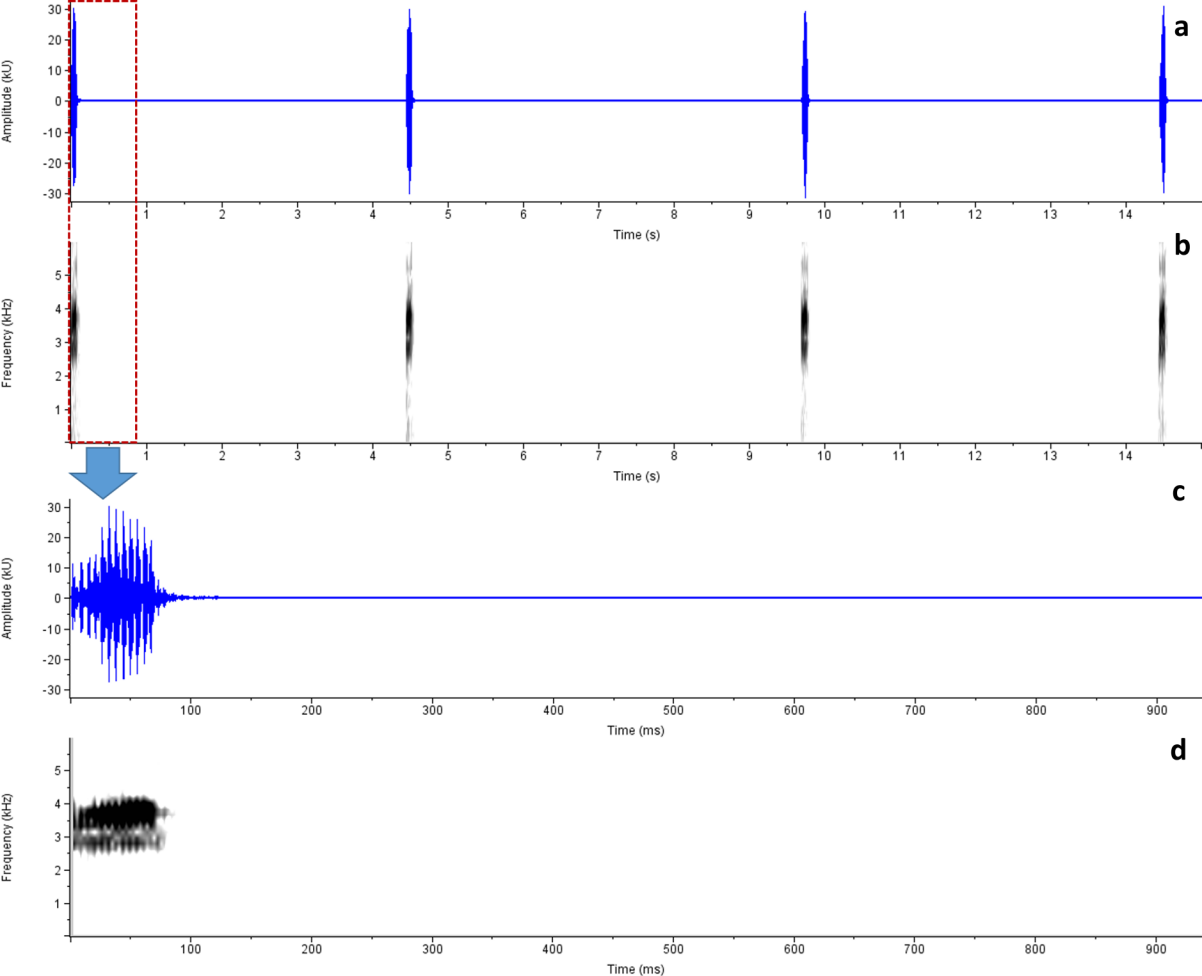
Advertisement call spectrogram of *Raorchestes griet*. a. Amplitude, b. Spectrogram, c. Amplitude of a single call and d. Spectrogram of a single call.

**Fig 8:**
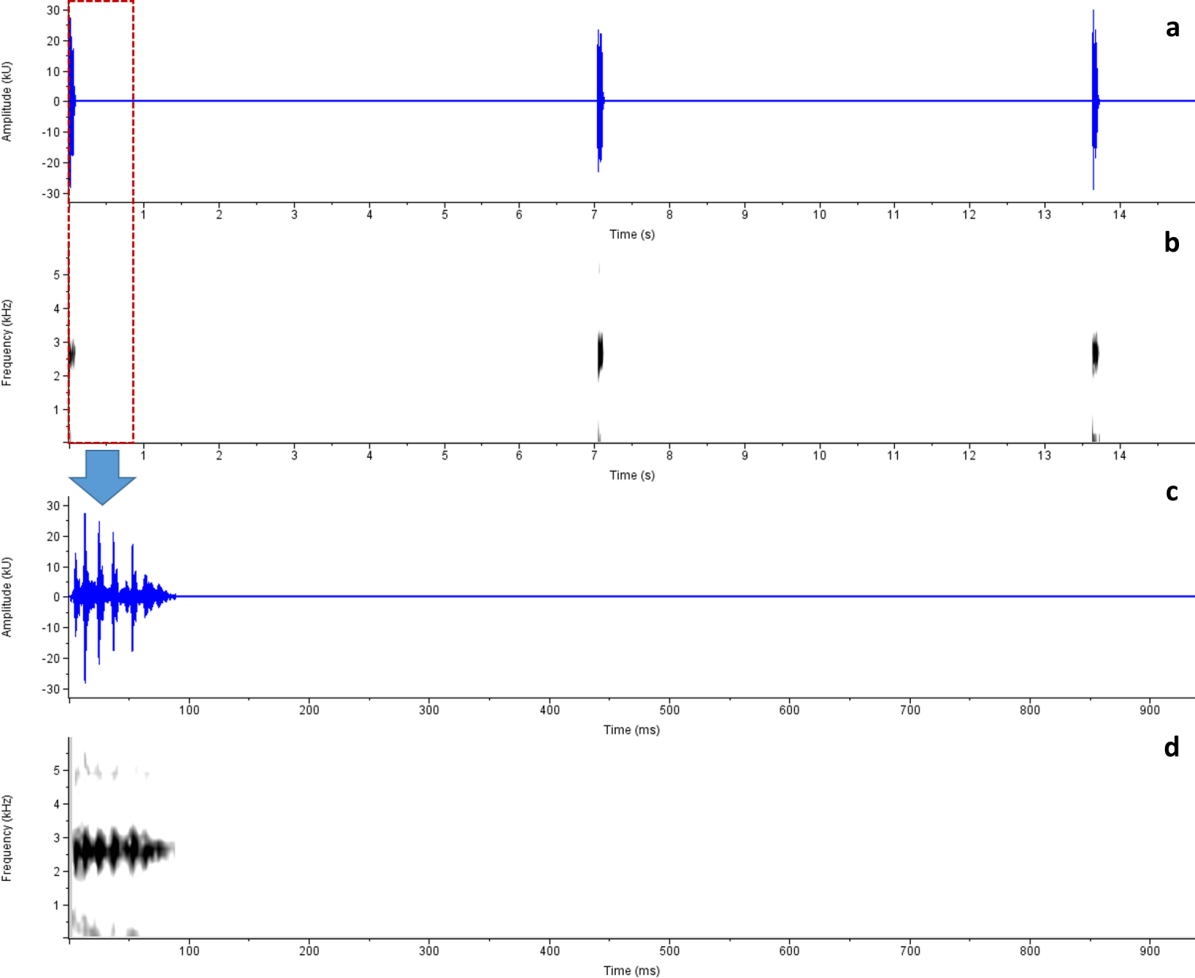
Advertisement call spectrogram of *Raorchestes honnametti* sp. nov. a. Amplitude, b. Spectrogram, c. Amplitude of a single call and d. Spectrogram of a single call.

*Advertisement call analysis*: Calls of *R*. *honnametti* were recorded on 13 October 2012, 20:15h, Air Temperature 18.03 ± 0.12°C, Relative Humidity 79 ± 1% at Honnametti, 11.8987° N, 77.1741° E, 1659 m amsl; calls of *R*. *charius* were recorded on 5 July 2013; 18:20hr; Air Temperature: 20.74 ± 0.21°C; Relative Humidity: 86.67 ± 1.16%, at Honey Valley,12.2312° N, 75.6558° E, 1033 m amsl and calls of *R*. *griet* were recorded on 29 August 2014; 19:30h at Munnar; Air Temperature and Relative Humidity not recorded. Advertisement calls of these species greatly resemble each other (Figs 6, 7 and 8; Tables 2 and 3). Phonetically, the advertisement call sounds like ‘*treenk…*..*treenk…*..*treenk…*’, however they vary in number of pulses, call duration, inter-call interval duration and dominant frequency. Table 2 provides detail on call characteristics of *R*. *honnametti*, *R*. *charius* and *R*. *griet*. Calls of *R*. *honnametti* had 6–9 pulses (Mean ± SE, 6.4 ± 0.18) in each call. Average dominant frequency was 2635.9 ± 11.75 Hz, Call duration was 0.1 ± 0.003 s, inter-call interval duration of a lone individual was 6.3 ± 0.31 s and in the presence of multiple calling individuals, inter-call interval duration was 2.45 ± 0.02 s. Calls of *R*. *charius* had 13–18 pulses in each call. Average dominant frequency was 2478.57 ± 12.56 Hz; Call duration was 0.09 ± 0.004 s; inter call duration was 2.51 ± 0.09 s in the presence of multiple calling individuals. Calls of *R*. *griet* had 13–15 pulses in each call. Average dominant frequency was 3699.88 ± 9.51 Hz, Call duration was 0.09 ± 0.0007 s, and inter-call interval duration was 5.22 ± 0.2 s in the presence of multiple calling individuals. Welch t-test showed significant differences in dominant frequency, call duration and number of pulses of all the three species (Table 3).

**Table 2:** Acoustic characteristics of *R*. *charius* (N = 21), *R*. *griet* (N = 8) and *R*. *honnametti* (N = 20).

Features	*R*. *charius*	*R*. *griet*	*R*. *honnametti*
**Dominant Frequency (Hz) Mean ± SE (Range)**	2478.57±12.56 (2346–2541)	3699.88±9.51 (3634–3746)	2635.9±11.75 (2536–2736)
**Call duration (s) Mean ± SE (Range)**	0.09±0.004 (0.06–0.14)	0.086±0.001 (0.081–0.089)	0.1±0.003 (0.006–0.121)
**Number of pulses Mean ± SE (Range)**	15.14±0.26 (13–18)	13.38±0.16 (13–15)	6.4±0.179 (6–9)
**Inter-call interval duration (s) Mean ± SE (Range)**	2.51±0.09 (1.88–3.47)	5.22±0.2 (4.31–6.82)	2.45±0.023 (2.32–2.55)
**Inter-call interval duration (s) with single male calling Mean ± SE (Range)**	-	-	6.3±0.31 (3.94–8.07)

**Table 3:** Welch t-test to compare means of call characteristics of *R*. *charius*, *R*. *griet* and *R*. *honnametti*. Value in parenthesis indicates level of significance (P).

	*R*. *charius* Dominant frequency	*R*. *charius* Call duration	*R*. *charius* Pulse #	*R*. *griet* Dominant frequency	*R*. *griet* Call duration	*R*. *griet* Pulse #
***R*. *griet* Dominant frequency**	-61.4468 (0.0001)					
***R*. *griet* Call duration**		0.1797 (0.8559)				
***R*. *griet* Pulse #**			4.7724 (0.0001)			
***R*. *honnametti* Dominant frequency**	-9.0426 (0.0001)			54.4185 (0.0001)		
***R*. *honnametti* Call duration**		-1.805 (0.078)			2.6189 (0.015)	
***R*. *honnametti* Pulse #**			27.4138 (0.0001)			21.7459 (0.00001)

*Natural history*: *Raorchestes honnametti* sp. nov. is known only from Biligiri Rangaswamy hills and is one of the very common frogs in that landscape. It is found in shola forests, evergreen forests, semi-evergreen forests and around human habitations. Individuals were found calling in an open area within *Ageratina adenophora* (Asteraceae) and *Strobilanthus* bushes. Some individuals were also found on tree saplings in the understory. Individuals call at a perched height between 0.48–1.00 m from ground. Call starts at around 6 pm and goes till early morning. During monsoon (June to September), individuals call almost throughout the day except on days with heavy rains or dry days. Other anuran species like *Pseudophilautus* sp., *Hylarana* sp., *Fejervarya* sp., *Duttaphrynus melanostictus*, *Microhyla sholigari*, *M*. *ornata*, *M*. *rubra* and *Euphlyctis cyanophlyctis* co-occur with *R*. *honnametti* in Biligiri Rangaswamy hills.

### Conservation

There are no immediate threats from human activities to this newly described species as Biligiri Rangaswamy hills is a tiger reserve and enjoys high level of protection. However, in the last one decade, a significant area of the Reserve has been taken over by highly invasive species like *Lantana camara* (Verbenaceae) and *Ageratina adenophora* (Asteraceae). On subsequent visits, we have seen several calling males of *R*. *honnametti* on *Lantana* and *Ageratina* bushes, indicating that this species might have adapted to the presence of these invasive species. However, a systematic research needs to be undertaken to assess the impact of invasive species on *R*. *honnametti*.

## Discussion

**Fig 9:**
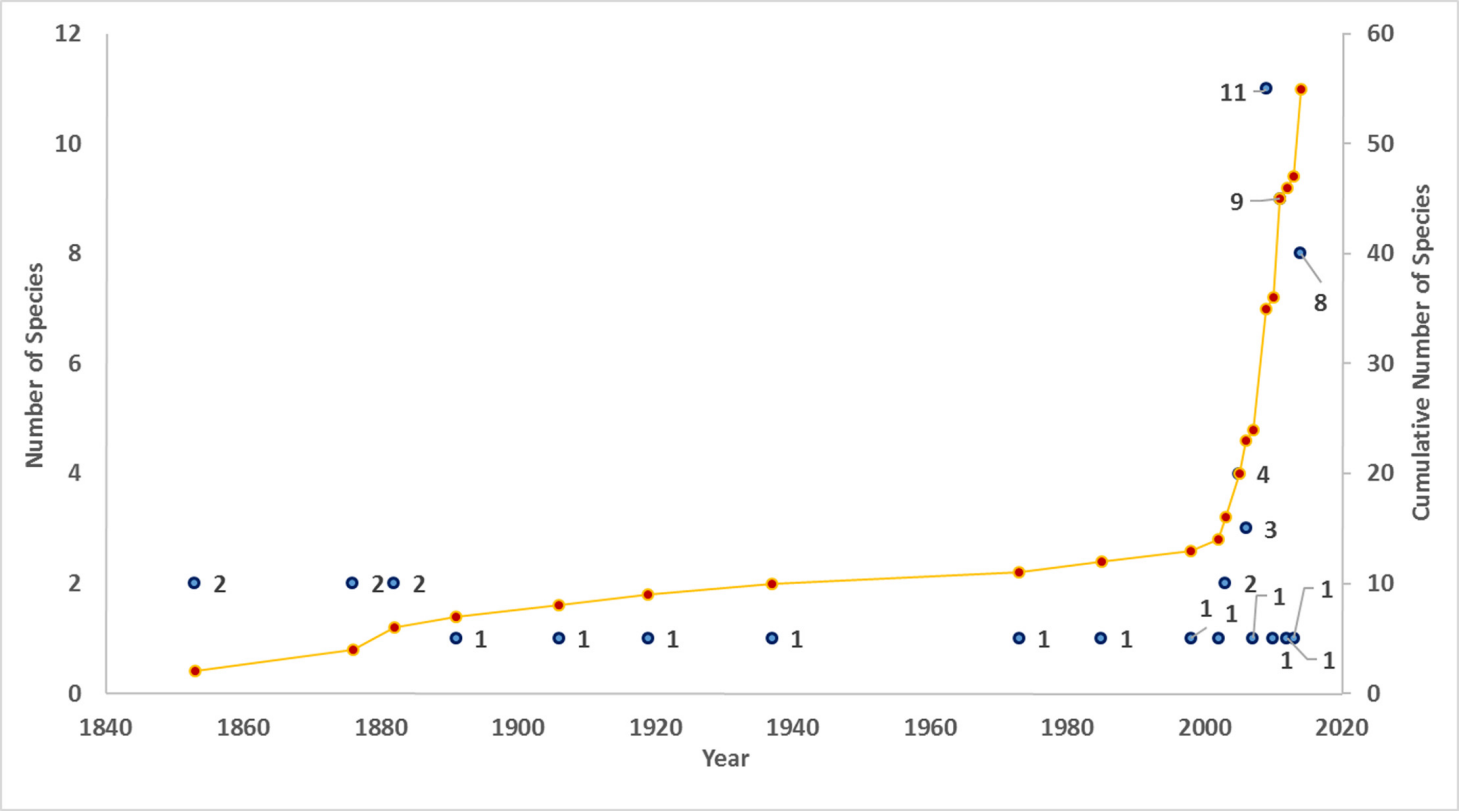
Species discovery pattern in the genus *Raorchestes* in India till 2014.

The bush frogs of the genus *Raorchestes* are distributed across India, southern China, Myanmar and Vietnam. India harbours 55 of these species, with 50 species distributed in the Western Ghats. With the addition of *R*. *honnametti* the total number of species in genus *Raorchestes* stands at 60. Discovery pattern shows that 42 species of *Raorchestes* were described in last 15 years (Fig 9), indicating that there may be many more species to be described from the Western Ghats and needs a continued systematic surveys of amphibians across the region. Such patterns in discovery further strengthen our expectations that not only morphologically distinct species, but also many cryptic species are waiting to be discovered from the Western Ghats.

Multivariate analysis of morphological data using LDA showed an overlap between *R*. *charius* and *R*. *honnametti* individuals while *R*. *griet* formed a distinct cluster. MANOVA and Tukey’s HSD post-hoc tests on morphological data did not show significant differences between *R*. *charius*, *R*. *griet* and *R*. *honnametti*, except for SL and TL. This validates our observations on morphological similarities between the three species and hence morphological characters alone cannot distinguish cryptic species. Tools like bioacoustics and molecular genetic technique can be a better aid in describing a cryptic species through integrated taxonomic approach. Studies have shown that a bioacoustic character like call dominant frequency has a highly significant negative relationship with body size in anurans [45]. A similar significant negative relationship was exhibited among the *Charius* clade members between dominant frequency (DF) and body size (SVL) (Pearson’s correlation coefficient, r = -0.74, p = 0.0004). External factors like temperature, relative humidity and altitude influence advertisement call characteristics [46]. Due to lack of data, we did not analyze the effect of external factors on advertisement call characteristics of *Charius* clade members.

Biju and Bossuyt [20] through their study on genus *Raorchestes* showed that *R*. *charius*, *R*. *griet* and *R*. *coonoorensis* formed a distinct clade. Recently, Vijayakumar et al [21] revised the *Charius* clade which involved only *R*. *charius* and *R*. *griet*, with *R*. *coonoorensis* forming a separate clade basal to the *Charius* clade. From our study, the *Charius* clade now includes *R*. *charius*, *R*. *griet* and *R*. *honnametti* with *R*. *charius* and *R*. *honnametti* forming sister taxa, while *R*. *griet* remains basal to the other two. The distribution range of these three species is restricted to separate regions of Western Ghats. According to the present distribution (Fig 1), *R*. *charius* is found in Bababudangiri to Brahmagiri hill ranges of central Western Ghats. It is 169 km north-west from *R*. *honnametti* and 251 km north from *R*. *griet* distribution. *R*. *griet* is found in Munnar and Valparai regions of southern Western Ghats below Palghat Gap. It is 169 km south west from *R*. *honnametti*, whereas *R*. *honnametti* (from this study) is found only in Biligiri Rangaswamy hills, south east of the Western Ghats. Absence of *R*. *charius* and *R*. *griet* in Biligiri Rangaswamy hills indicates that each of the three species has a smaller non-overlapping geographical range.

**Fig 10:**
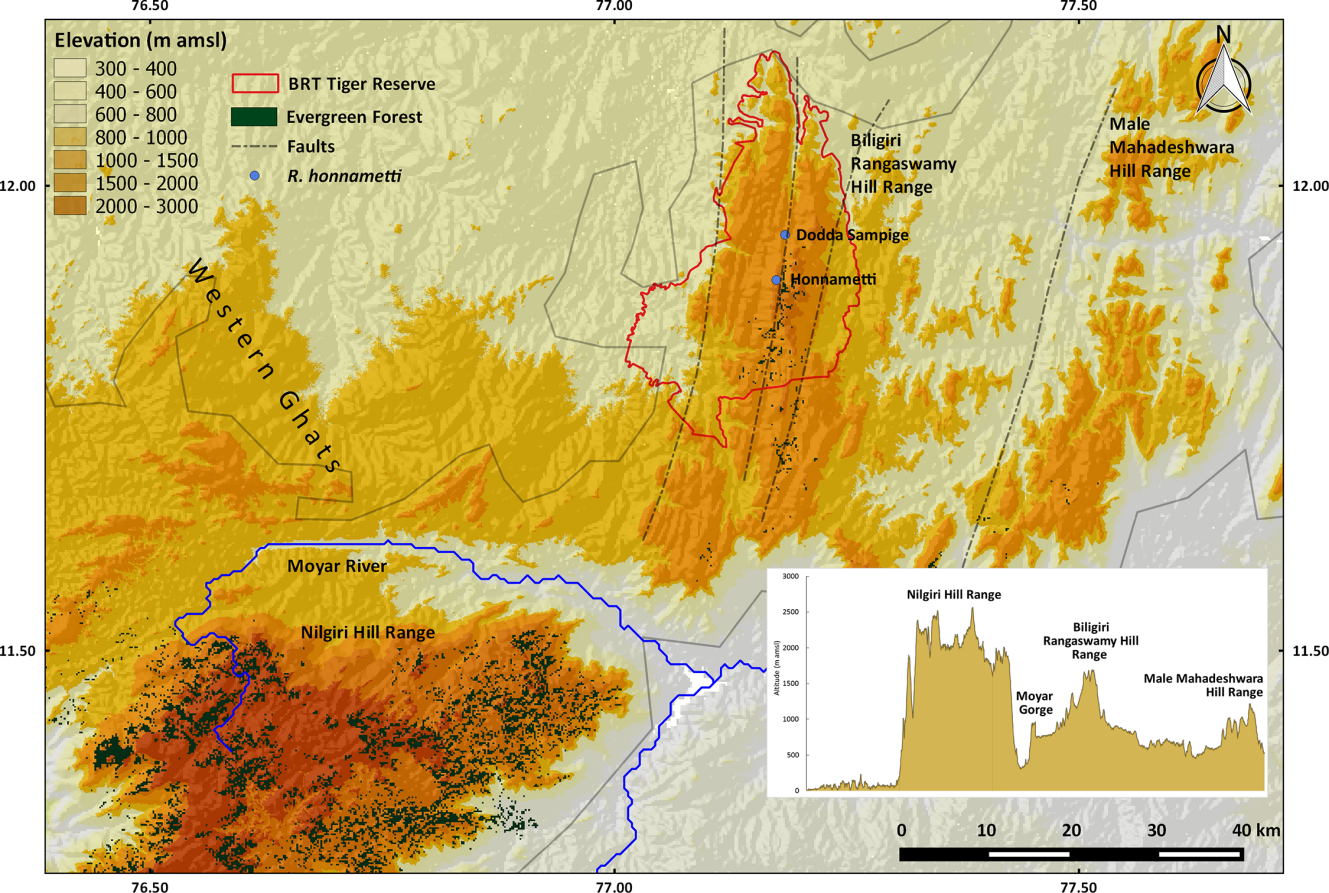
Elevation profile of hill ranges in and around BRT Tiger Reserve. Faults within Biligiri Rangaswamy hill ranges are shown with dash lines. Maps and elevation profile were generated using QGIS® Pisa Ver. 2.10. Data was sourced from www.gadm.org for administrative boundary of India and Shuttle Radar Topography Mission (SRTM) 90 m database (http://srtm.csi.cgiar.org) for elevation.

Studies understanding cryptic speciation signify the need to understand role of historical and biogeographical processes. The phenomenon of morphological stasis and genetic divergence in cryptic species could be because of recent speciation events owed to climatic oscillations and geographic barriers [47]. Biju and Bossuyt [20], stated that species of genus *Raorchestes* showed a high level of local endemism and emphasized the role of isolated hilly regions as a major reason for restricted species distribution. Studies understanding the divergence in frog lineages indicate that not just mountains but ecology, climatic conditions, forest types and rivers can act as barriers [48, 49, 50]. *Raorchestes honnametti* is a nocturnal, arboreal and a direct developing frog. Though detailed ecological study on *R*. *honnametti* is warranted, above mentioned ecological factors could have acted as barriers resulting in speciation of *R*. *honnametti*. The geological studies on Biligiri Rangaswamy hills suggest that it is a horst mountain formed due to the movement of Kollegal fault running N-NE to S-SW direction (Fig 10) and might have formed recently during late Quaternary period (1.80 to 2.58 Ma) along the eastern borders of Mysore Plateau [51]. The underlying rocks date back to 3.6 Ga of Dharwad Craton. This uplift of Biligiri Rangaswamy hills–Male Mahadeshwara hills and subsequent isolation of Biligiri Rangaswamy hills from Niligiris due to Moyar Gorge could be one of the reasons for diversification of *R*. *honnametti* from *R*. *charius* and *R*. *griet*.

The restricted distribution of *R*. *honnametti* could also be due to the present day disjunct distribution of evergreen forests in Biligiri Rangaswamy hills. Studies have shown that such forests of India got restricted due to variation in climatic conditions (Miocene and Quaternary period) and anthropogenic factors resulting in a disjunct distribution of species [52]. Molecular dating can further help in understanding the diversification in *R*. *honnametti* as well its other clade members. Nevertheless, the discovery of *R*. *honnametti* opens up interesting evolutionary questions on cryptic species and studies understanding ecological and evolutionary mechanisms can help in appreciating the conserved morphology of such cryptic species.

## Supporting Information

S1 Table GenBank accession numbers for 16S rRNA gene of 49 species of *Raorchestes* and *Rhacophorus malabaricus* compared in the study. (XLSX)

S2 Table GenBank accession numbers for ND1 gene of 49 species of *Raorchestes* and *Rhacophorus malabaricus* compared in the study. (XLSX)

S3 Table Details of type materials and voucher specimens examined. (XLSX)

S4 Table Genetic divergence (in percentage) matrix of 50 species of *Raorchestes* and an outgroup *Rhacophorus malabaricus*. (XLSX)

S5 Table Linear Discriminant Analysis factor loadings for Axis 1 and Axis 2. (XLSX)

S6 Table Morphological comparison of *R*. *honnametti* with *R*. *thodai* and with non-Western Ghats species of *Raorchestes*. (DOCX)
