# Supplementary material for: Integrative Taxonomic Approach for Describing a New Cryptic Species of Bush Frog (Raorchestes: Anura: Rhacophoridae) from the Western Ghats, India
Source: PLoS One. 2016 Mar 2;11(3):e0149382. doi: 10.1371/journal.pone.0149382 (PMC4774957; doi:10.1371/journal.pone.0149382)
Supplement: S6 Table — (DOCX) [file pone.0149382.s006.docx]

**S6 Table.** Morphological comparison of *R. honnametti* with *R. thodai* and with *Raorchestes* species non-endemic to the Western Ghats.

**Comparison with *R. thodai***

Morphologically *R. thodai* is distinct from *R. honnametti* in following characters: adult size (*R. thodai*: 33.7–34 mm vs *R. honnametti*: 21.7–24.8 mm), snout length (*R. thodai*: 5.2–6.0 mm vs. *R. honnametti*: 3.1–3.9 mm), snout (*R. thodai*: pointed vs. *R. honnametti*: oval), lingual papilla (*R. thodai*: present vs. *R. honnametti*: absent), dermal fringes (*R. thodai:* present on fingers and toes in vs. *R. honnametti*: absent) and groin color (*R. thodai*: groin cream white without any yellow blotches vs. *R. honnametti*: groin brown with 3–4 yellow blotches).

**Comparison with non-Western Ghats species of *Raorchestes***

*Raorchestes* *annandalii* differs from *R. honnametti* in following characters: adult size (*R. annandalii*: 16 mm vs *R. honnametti*: 21.7–24.8 mm), snout (*R. annandalii*: pointed vs. *R. honnametti*: oval), nostril from tip of snout (*R. annandalii*: equidistant from snout and eye vs. *R. honnametti*: closer to tip of snout), discs on fingers and toes (*R. annandalii*: not well developed vs. *R. honnametti*: well developed) and skin on dorsum (*R. annandalii*: smooth vs. *R. honnametti*: with horny spines).

*Raorchestes* *manipurensis* differs from *R. honnametti* in following characters: adult size (*R. manipurensis*: 25 mm vs *R. honnametti*: 21.7–24.8 mm), snout (*R. manipurensis*: obtusely pointed vs. *R. honnametti*: oval), tympanum (*R. manipurensis*: distinct, 2 mm vs. *R. honnametti*: indistinct, 1.4 mm), webbing between fingers (*R. manipurensis*: rudimentary vs. *R. honnametti*: absent), webbing between toes (*R. manipurensis*: well developed vs. *R. honnametti*: moderate), skin on dorsum (*R.* *manipurensis*: smooth vs. *R. honnametti*: with horny spines) and color (*R.* *manipurensis*: venter dirty white, speckled with brown vs. *R. honnametti*: Venter uniform cream white, vocal sac yellow translucent with granular grey spots).

*Raorchestes* *shillongensis* differs from *R. honnametti* in following characters: adult size (*R. shillongensis*: 10–20 mm vs *R. honnametti*: 21.7–24.8 mm), snout (*R. shillongensis*: pointed vs. *R. honnametti*: oval), belly (*R. shillongensis*: with numerous warts vs. *R. honnametti*: granular) and longitudinal fold (*R. shillongensis*: from axilla to foreparts of thigh vs. *R. honnametti*: absent).

*Raorchestes* *sahai* differs from *R. honnametti* in following characters: adult size (*R. sahai*: 25–26 mm vs *R. honnametti*: 21.7–24.8 mm), nostril from tip of snout (*R. sahai*: equidistant from snout and eye vs. *R. honnametti*: closer to tip of snout), tympanum (*R. sahai*: distinct, 1.5 mm vs. *R. honnametti*: indistinct, 1.4 mm), webbing between toes (*R. sahai*: well developed vs. *R. honnametti*: moderate) and color (*R.* *sahai*: venter dull white with light-darker blotches on the belly vs. *R. honnametti*: venter uniform cream white, vocal sac yellow translucent with granular grey spots).

*Raorchestes* *terebrans* differs from *R. honnametti* in following characters: adult size (*R. terebrans*: 12.8–21.6mm vs *R. honnametti*: 21.7–24.8 mm), snout (*R. terebrans*: obtusely pointed vs. *R. honnametti*: oval), metatarsal tubercle (*R. terebrans*: both inner and outer metatarsal tubercles present vs. *R. honnametti*: only inner metatarsal tubercle present) and color (*R.* *terebrans*: throat brownish vs. *R. honnametti*: throat cream white with grey spots).

*Raorchestes* *gryllus* differs from *R. honnametti* in following characters: adult size (*R. gryllus*: 25-27 mm vs *R. honnametti*: 21.7–24.8 mm), snout (*R. gryllus*: pointed in males vs *R. honnametti*: oval in males), tympanum (*R. gryllus*: distinct, big vs *R. honnametti*: indistinct), nuptial pad (*R. gryllus*: present vs *R. honnametti*: absent) webbing between fingers (*R. gryllus*: rudimentary, between 3^rd^ and 4^th^ finger vs. *R. honnametti*: absent), heel (*R.* *gryllus*: with a small pointed appendage vs. *R. honnametti*: no appendages), skin (*R.* *gryllus*: smooth above, except on upper eyelid and head vs. *R. honnametti*: entire dorsum with small horny spines) and dermal fringe (*R. gryllus*: along forelimb and tarsus well developed vs *R. honnametti*: absent).

*Raorchestes* *parvulus* differs from *R. honnametti* in following characters: adult size (*R. parvulus*: 23.6 mm vs *R. honnametti*: 21.7–24.8 mm), dermal fringe on fingers (R. *parvulus*: present on all fingers vs. *R. honnametti*: absent), skin on dorsum (*R.* *parvulus*: smooth vs. *R. honnametti*: with small horny spines) and color (*R.* *parvulus*: venter brown or whitish with brown mottling vs. *R. honnametti*: Venter uniform cream white, vocal sac yellow translucent with granular grey spots).

*Raorchestes* *longchuanensis* differs from *R. honnametti* in following characters: adult size (*R. longchuanensis*: 21.9 mm [SVL measured from holotype available online from Kunming Natural History Museum of Zoology [34]] vs *R. honnametti*: 21.7–24.8 mm), disc color (*R.* *longchuanensis*: orange/red colored vs. *R. honnametti*: grey/cream colored) and webbing between toes (R. *longchuanensis*: reduced vs. *R. honnametti*: moderate).

*Raorchestes* *menglaensis* differs from *R. honnametti* in following characters: adult size (*R. menglaensis*: 16–20 mm vs *R. honnametti*: 21.7–24.8 mm), snout (*R.* *menglaensis*: acutely pointed vs. *R. honnametti*: oval) and color (R. *menglaensis*: cream white at groin without yellow blotches vs. *R. honnametti*: groin uniform light brown with 3–4 yellow blotches).

**Comparison with synonyms for available name**

*Raorchestes emeraldi, R. neelanethrus* and *R. montanus were* synonymised with *R. flaviventris, R. luteolus* and *R. hassanensis* respectively [20, 39]. However, to ensure whether the earlier names are available for the new species, we made morphological comparisons of synonyms with *R. honnametti*.

*Raorchestes neelanethrus* differs from *R. honnametti* in following characters: adult size (*R. neelanethrus*: 21.4–29.9 mm vs *R. honnametti*: 21.7–24.8 mm), snout (*R.* *neelanethrus*: pointed vs. *R. honnametti*: oval), supratympanic fold (*R.* *neelanethrus*: indistinct vs. *R. honnametti*: distinct), color (R. *neelanethrus*: body yellow without yellow blotches in groin vs. *R. honnametti*: body grey and groin uniform light brown with 3-4 yellow blotches), dominant frequency (R. *neelanethrus*: 2350 Hz vs. *R. honnametti*: 2635.9 ± 11.75 Hz)

*Raorchestes emeraldi* differs from *R. honnametti* in following characters: adult size (*R. emeraldi*: 36.5–50.5 mm vs *R. honnametti*: 21.7–24.8 mm), tympanum (*R. emeraldi*: large, distinct 2.2mm vs. *R. honnametti:*  indistinct 1.4 mm), lingual papilla (*R. emeraldi*: present vs. *R. honnametti: absent*), dorsum color (*R.* *emeraldi*: green with yellow spots vs. *R. honnametti*: grey) and color in groin (R. *emeraldi*: brown and yellow reticulated pattern in the regions of groin, front and back of thighs, underside of shank and front of metatarsal vs. *R. honnametti*: groin uniform light brown with 3–4 yellow blotches).

*Raorchestes montanus* differs from *R. honnametti* in following characters: adult size (*R. montanus*: 37 mm vs *R. honnametti*: 21.7–24.8 mm), lingual papilla (*R. montanus*: present vs. *R. honnametti: absent*), webbing between toes (*R. montanus*: 2/3^rd^ webbed vs. *R. honnametti*: moderate), skin on dorsum (R. *montanus*: smooth vs. *R. honnametti*: with small horny spines) and color in groin (*R.* *montanus*: without yellow blotches vs. *R. honnametti*: uniform light brown with 3–4 yellow blotches).

Hence, these three synonym names are not available for the new species.
